# Soybean Peroxidase Catalyzed Decoloration of Acid Azo Dyes

**DOI:** 10.5696/2156-9614-10.25.200307

**Published:** 2020-02-28

**Authors:** Bahaa Malik Altahir, Teeba Jaffar Al-robaiey, Zainab Mohammad Abbaas, Neda Mashhadi, Laura G. Cordova Villegas, Keith E. Taylor, Nihar Biswas

**Affiliations:** 1 Biology Department, College of Science, University of Baghdad, Baghdad, Iraq; 2 Department of Chemistry and Biochemistry, University of Windsor, Windsor, Ontario, Canada; 3 Department of Civil and Environmental Engineering, University of Windsor, Windsor, Ontario, Canada (cordoval@uwindsor.ca)

**Keywords:** azo dye, acid dyes, Acid Black 2, Acid Orange 7, decoloration, peroxidase

## Abstract

**Background.:**

Some industrial manufacturing processes generate and release dyes as water pollutants, many of which are toxic and hazardous materials. There is a need for milder, greener methods for dye treatment.

**Objectives.:**

The objective of the present study was to investigate and optimize azo dye decoloration by a crude soybean peroxidase (SBP), based on two dyes that have widespread industrial use, but that differ greatly in structural complexity, Acid Black 2 and Acid Orange 7, and to investigate the effects of specific parameters on the removal process.

**Methods.:**

Batch reactors were used to remove 95% of the dyes' color and to produce substantial precipitates.

**Results.:**

The optimum pH for enzymatic decoloration of Acid Black 2 was in the acidic region, pH 4.4, and that of Acid Orange 7 occurred under neutral conditions, pH 6.9. The minimum enzyme activity needed for sufficient removal was 1.2 U/mL for both dyes at 0.5 mM. The minimum molar hydrogen peroxide/substrate ratio was 3 for Acid Orange 7 and 2.5 for Acid Black 2 to achieve approximately 95% removal. First-order fitting of progress curve data collected under the respective optimum conditions gave half-lives of 23.9 and 28.9 minutes for Acid Orange 7 and Acid Black 2, respectively.

**Conclusions.:**

The feasibility of SBP-catalyzed treatment of industrial dyes Acid Black 2 and/or Acid Orange 7, or dyes that resemble them, as they might occur in industrial effluents, was successfully demonstrated.

**Competing Interests.:**

The authors declare no competing financial interests

## Introduction

Synthetic dyes are composed of stable chemical compounds which are commonly released into wastewater and represent a serious water pollution issue in the textile industry.^1^

**Figure 1:**
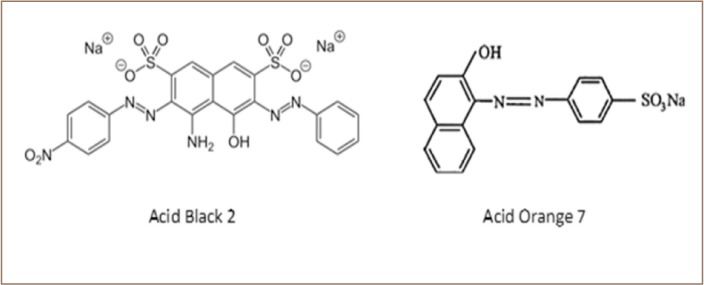
Chemical structures of Acid Orange 7 and Acid Black 2 dyes

In recent decades, tremendous development in the industrial and agricultural fields has led to the use of chemical compounds that are released into the environment in the form of toxic contaminants.^2^ Dyes are among these compounds, used in many industries such as furniture manufacturing, textiles, food production and paint.^3^ Thousands of dyes were developed and used at the beginning of the 20th century.^4^ The presence of low concentrations of dyes in aqueous solutions can be toxic.^5^ In some situations, more than 15% of the transferable dyes are lost to surface water. 4 Some compounds that are produced from partial decomposition of dyes, such as aniline, are carcinogenic, toxic and mutagenic.^6^ Some of the major causes of environmental risk are related to the recalcitrance of dyes to biodegradation.^7^ Two azo dyes were chosen for this study, Acid Black 2 and Acid Orange 7 *(Figure 1 and Table 1).* Acid Orange 7 is a simple monoazo dye, found in wastewater of cosmetic and textile industries.^8^ Acid Black 2 is a structurally more complex, bisazo dye used to make India ink.^9^

**Table 1:** General Characteristics of Acid Orange 7 and Acid Black 2 25, 26

	**Molecular formula**	**Color index**	**Molecular weight (g mol^−1^)**	**Water solubility (gL^−1^)**
Acid Orange 7	HOC_10_H_6_N=NC_6_H_4_SO_3_Na	15510	350	116
Acid Black 2	C_48_H_36_N_9_	50420	739	50

Various degradation procedures for dyes have been reported in the literature. Physical methods often used include sonophotolysis, semiconductor photo-catalysis, microwave methods, non-thermal plasma processing, photocatalytic-degradation and adsorption.^2^,^3^,^10–14^

Various chemical procedures have been employed, such as electro-chemical oxidation, reaction with coated and treated metal oxides, Fenton and photo-Fenton processes, and other advanced oxidation processes using different catalysts.^3^,^5–8^,^14–22^

Biological treatments have also been reported in the literature, such as bacterial decolorization, aerobic biological treatment, cyanobacteria reactivity, and fungal decolorization.^1^, ^4^, ^21^, ^23^, ^24^

Incomplete color removal, cost and/or production of secondary pollutants are disadvantages of these methods.^12^ In addition, the generation of hazardous by-products, large amounts of sludge and low efficiency are problems associated with conventional treatment methods.^27^

A new method based on the use of enzymes such as peroxidases was developed by Klibanov *et al.* in 1980.^28^ During the last decades, the application of enzymatic treatments underwent great development such as identifying new substrates and various peroxidases in addition to understanding mechanisms of reaction and inactivation, the effect of additives and different reactor configurations.^29^ Reaction under mild conditions and at high reaction rates are the main advantages of dye degradation using enzymes.

Some azo dyes have been decolorized by using peroxidases such as soybean peroxidase (SBP), lignin peroxidase, manganese peroxidase, and horseradish peroxidase, as well as laccases.^30^ It has been suggested that enzymatic treatment could oxidize the dye structures to form compounds with lower toxicity and may eventually mineralize the dyes through enzymatic radical formation, and subsequent removal.^31^

Previous studies have examined chemical and enzymatic decoloration, but few have dealt with the use of inexpensive crude enzymes such as SBP in the decoloration of acid dyes. Only one recent report used the same SBP preparation as the present study, which highlights the need for continued exploration of the scope for enzymatic azo dye treatment in order to arrive at a generalized treatment process.^32^ Soybean seed coat peroxidase is well suited for this study because of its stability, ease of extraction, widespread availability and potential for adding to the soy value chain.^33^

**Abbreviations**
*H*_2_*O*_2_: Hydrogen peroxide
*SBP*: Soybean peroxidase

The present study evaluated the feasibility of SBP catalyzed oxidative dye polymerization and removal of Acid Orange 7 and Acid Black 2.

## Methods

Crude dry solid SBP (Enzyme Commission number 1.11.1.7, industrial grade, lot number 18541NX, Reinheitszahl value of 0.75 ±0.10) was obtained from Organic Technologies (Coshocton, OH, USA). Dry solid bovine liver catalase (Enzyme Commission number 1.11.1.6, lot number 120H7060, 19 900 U/mg solid) was purchased from Sigma-Aldrich Chemical Company Inc. (St. Louis, MO, USA). Acid Black 2, i.e. Nigrosin water soluble, and Acid Orange 7, i.e. Orange II sodium salt, were purchased from Sigma-Aldrich Chemical Company. The enzymes were stored at −15°C, and their sub-stock solutions (70 U/mL for SBP and 17000 U/mL for catalase) were stored at −4°C. Hydrogen peroxide (30% wt/vol) was provided from ACP Chemicals, Inc. (Montreal, Quebec) and stored at 4°C. All other chemicals were of highly pure or analytical grade purchased from Sigma-Aldrich or BDH Chemical Corporation, Inc., (Toronto, Ontario). The spectrophotometer used was a Carey 100, Varian Australia.

### 

#### Soybean peroxidase activity assay

A spectrophotometric procedure was used to measure free SBP activity. Briefly, this assay determines the rate of color formation at 510 nm due to the quinone imine formed in the oxidative coupling of phenol and 4-aminoantipyrine catalyzed by SBP in the presence of hydrogen peroxide.^34^ The assay utilized a reagent consisting of 10 mM phenol, 40 mM phosphate buffer (pH 7.4), 2.4 mM 4-aminoantipyrine, and 0.2 mM hydrogen peroxide (H_2_O_2_) in a total volume of 950 μL.^27^ The reaction was then started by adding 50 μL of the diluted enzyme solution to 0.95 mL of the reagent, and the initial rate of color formation at 510 nm was monitored for 30 seconds. The sample dilution was adjusted according to the expected enzyme activity (to have approximately 0.2 absorbance change in 30 seconds).^35^ One unit of SBP activity is defined as the amount catalyzing 1 μmol of hydrogen peroxide conversion per minute under the assay conditions.^29^

#### Dye assays

Since the dyes absorb in visible wavelengths, all the samples were examined by direct spectrophotometric determination at 605 nm for Acid Black 2 and 510 nm for Acid Orange 7.

#### Buffer preparation

Buffer preparation was accomplished according to the method outlined by Stoll and Blanchard.^36^ Acetate buffer with a pH range of 3.6–5.6, phosphate buffer with a pH range of 5.7–8.0, and carbonate-bicarbonate buffer with a pH range of 9.2–10.7 were used to obtain the proper pH control.

#### Experimental protocol

Batch reactors were used for Acid Black 2 and Acid Orange 7 to remove 95% of the substrate. The parameters that were optimized were pH, enzyme activity and hydrogen peroxide concentration. All experiments were conducted at room temperature in 20-mL glass flasks with 0.5 mM substrate. The buffer used was acetate, carbonate or phosphate (40 mM) to cover the pH range of 3.5 to 8.0. The stirring time was 3 hours using a magnetic stirrer and Teflon-coated magnetic stir bars for all experiments at room temperature.^27^ The reaction time was chosen to be consistent with previous studies of 95% phenol and aniline degradation.^33^,^35^,^37^ The optimal conditions to remove 95% of these dyes were determined. This degree of removal was chosen because 5% remaining can be detected with high precision, while the range of 1–4% remaining would be close to or below the detection limit of the analytical procedure employed.

The components of the sample mixture were added at the appropriate concentrations in the following order to initiate the reaction: water, buffer, dye, SBP and hydrogen peroxide. Reactions were stopped by adding excess catalase to 17 U/mL final concentration to quench the residual hydrogen peroxide by quick consumption.^27^

**Figure 2:**
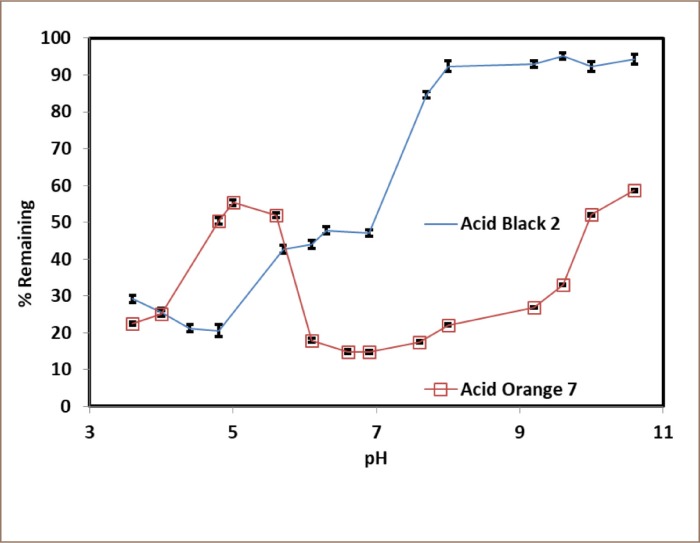
pH optimization for Acid Black 2 and Acid Orange 7. Hydrogen peroxide and substrates were equimolar (0.5 mM), the enzyme activity was 0.5 U/mL, the reaction time was 3 hours.

**Figure 3:**
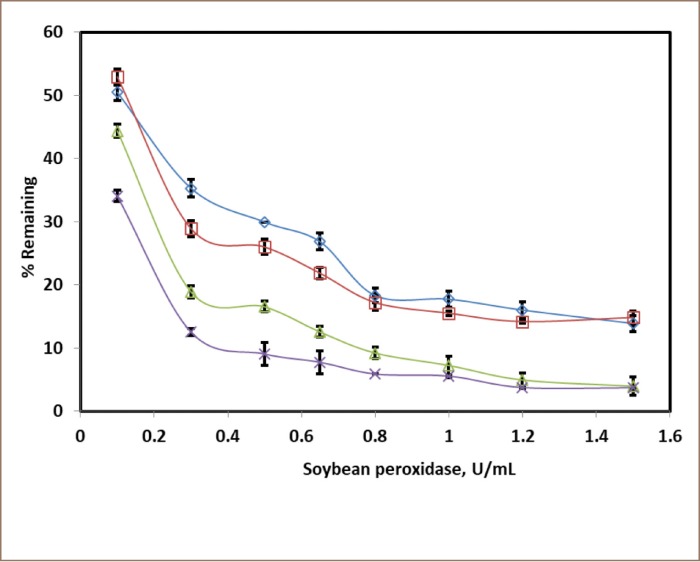
Soybean peroxidase optimization experiments for color removal. Dyes at 0.5 mM, for 3 hours, with conditions: 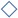, 0.5 mM H_2_O_2_ at pH 6.9 for Acid Orange 7; 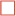, 0.5 mM H_2_O_2_ at pH 4.4 for Acid Black 2; 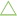, 0.65 mM H_2_O_2_ at pH 6.9 for Acid Orange 7; 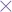, 0.65 mM H_2_O_2_ at pH 4.4 for Acid Black 2.

**Figure 4:**
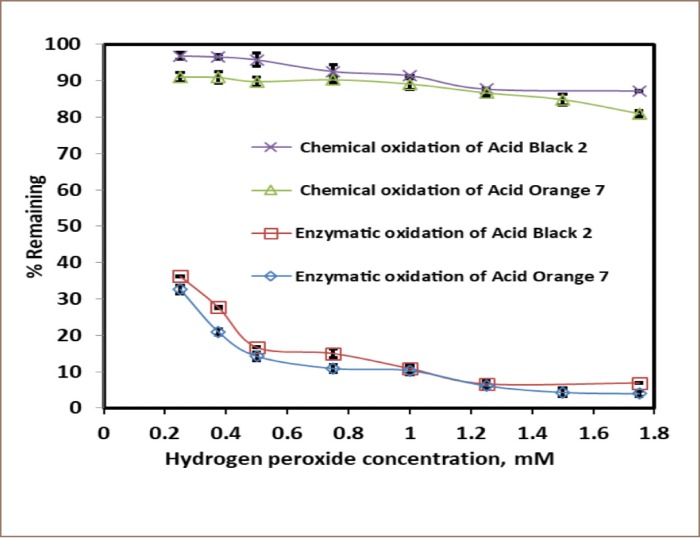
Effect of the H_2_O_2_ concentration on color removal. Dyes at 0.5 mM, pH 4.4 for Acid Black 2 and pH 6.9 for Acid Orange 7 with or without 1.2 U/mL SBP and reaction time of 3 hours.

**Figure 5:**
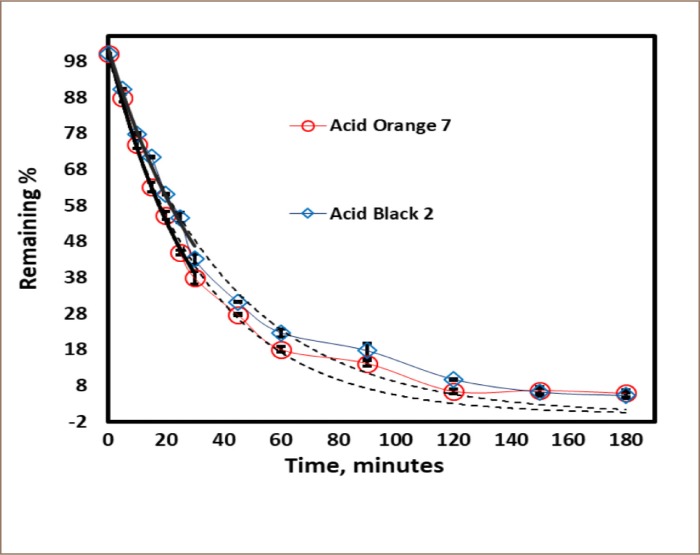
Color removal progress curves. Under optimal conditions: 0.5 mM dyes at the optimum pH (6.9 for Acid Orange 7 (red trend line) and 4.4 for Acid Black 2 (blue trend line)), enzyme activity of 1.2 U/mL SBP, hydrogen peroxide concentration 1.50 mM for Acid Orange 7 and 1.25 mM for Acid Black 2.

The samples were then centrifuged and micro-filtered using a 0.45-μm micro-syringe filter before spectrophotometric determination.^29^ All experiments were conducted in triplicate under the same conditions and the corresponding standard deviations were calculated and are represented by the error bars provided in Figures 2–5.

Time course studies, in 200 mL batch reactors, with 0.5 mM substrate (Acid Orange 7 or Acid Black 2), were carried out under the optimal conditions of pH, minimum hydrogen peroxide and enzyme concentrations. Aliquots (5 mL) were withdrawn by micropipette at various time intervals, quenched with 0.25 mL catalase solution and then centrifuged, micro-filtered and analyzed by a spectrophotometer. Centrifugation was used for quick sample preparation in order to get a clear liquid sample.^35^

## Results

Qualitatively, both dye reactions produced precipitates and the solutions were decolorized. The Acid Orange 7 solution was converted to a pale yellow from deep orange with small yellowish precipitate particles, while the Acid Black 2 solution was converted to a colorless one with larger blue precipitate particles. Centrifugation and microfiltration were conducted for all samples before spectrophotometric determination to remove any interference during measurement. Studies of the most important parameters, pH, enzyme activity, hydrogen peroxide concentration and time course were performed in completely-mixed batch reactors. Such information would allow the design of a treatment system and prototype reactor. Enzyme immobilization, often considered for enzymatic treatment systems, was not a viable option here because of the formation of solid products.

### Optimization of dye decoloration from water

The experiments were designed to test the effect of pH under stringent conditions with respect to enzyme activity (insufficient activity to achieve complete conversion of substrate) to clearly accentuate the pH effect, as seen in Figure 2. The optimum pH for the enzymatic conversion of Acid Black 2 was in the acidic range (4.3–4.8), while the optimum pH for Acid Orange 7 covered a broad range of neutral conditions (6.0–8.0).

### Enzyme activity

The successive increase in the activity of the SBP at the respective optimal pH values was studied over 3 hours *(Figure 3)*. The goal of the experiments was to achieve 95% removal of dyes. As seen, equimolar hydrogen peroxide was insufficient to achieve 95% removal with the range of enzyme activity 0.1–1.5 U/mL (compare Figure 3 symbols 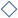 with 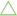 and 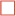 with 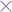). Figure 3 shows that the minimum SBP activity required for 95% conversion of dyes was 1.2 U/mL, which was chosen as the minimum effective enzyme activity for subsequent experiments.

### Optimum H_2_O_2_-to-substrate concentration ratio

Using previously established optimal enzyme activity and pH, molar concentrations of H_2_O_2_ in the range 0.25–1.75 mM were studied for the enzymatic reaction of 0.5 mM Acid Orange 7 and Acid Black 2 over 3 hours with the goal of 95% conversion *(Figure 4).* Chemical oxidation (without enzyme) was minimal over the same peroxide concentration range. The minimum hydrogen peroxide/substrate molar ratio required to achieve 95% removal was 3.0 for Acid Orange 7 and 2.5 for Acid Black 2.

#### Effect of reaction time

The time courses for the enzymatic reactions of the dyes were determined at the respective optima for pH, enzyme activity and minimum hydrogen peroxide concentration. The progress curves, red and blue trend lines in Figure 5, show approximately 95% removal in 3 hours. For quantitative analysis, the data were fit to a pseudo-first-order model for both molecules, as indicated by the black lines drawn in Figure 5. The black solid lines represent the first 30 minutes and the black dashed lines signify the entire time. The data were fitted to: percentage remaining = (initial percentage) e^−kt^, where the fitted value of the initial percentage should be close to 100% and k is the apparent first-order rate constant *(Table 2)*.

**Table 2:** Parameter Fit of Progress Curves for Color Removal of Acid Orange 7 and Acid Black 2

	**k, min^−1^**	**Half-life, min**	**Initial percentage %**	**R^2^**	**Represented in Figure 5**
**First 30 min**					

Acid Orange 7	(3.2 ±0.1) ^× 10−2^	21.7	102 ± 1.1	0.9963	Solid black line
Acid Black 2	(2.6 ±0.1) ^× 10−2^	26.7	102 ± 1.7	0.9873	Solid black line
**Whole time**					

Acid Orange 7	(2.9 ±0.1) ^× 10−2^	23.9	99.4 ± 2.4	0.9892	Dashed black line
Acid Black 2	(2.4 ±0.1) ^× 10−2^	28.9	100 ±2.3	0.9893	Dashed black line

Abbreviations: min, minute; R^2^, correlation coefficient; k, rate constant.

Thus, fitting the entire time course (180 minutes) was only slightly less precise, giving lower rate constants by approximately 10% for Acid Orange 7 and 9% for Acid Black 2, respectively, with corresponding lower half-lives of 23.9 and 28.9 minutes.

## Discussion

The current method is compared to other non-enzymatic peroxide-based methods for dye removal in Table 3 and compared to other enzymatic dye methods based on SBP in Table 4.

**Table 3:** Comparison of Current Enzymatic Method with Other Non-Enzymatic Hydrogen Peroxide-Based Methods

**Method**	**pH**	**[H_2_O_2_] mM/[substrate] mM**	**Rate constant**	**Reference**
**Acid Orange 7**				

Fenton	7	266.7/0.14	-	38
Ozone/H_2_O_2_	6	0.23/0.227	k=1.5×10^−2^ min^−1^	26
Homogeneous Fenton	4	2/0.085	-	39
Heterogeneous Fenton	3	7.77/0.227	-	40
Heterogeneous Fenton/ultrasonic	3	4/0.28	k=(7 ± 0.001)×10^−2^ min^−1^	41
Proposed method	6.9	1.5/0.5	k=(3.2 ± 0.08)×10^−2^ min^−1^	________
**Acid Black 2**				

Solar Photo-Fenton	3–4	9.77/0.1	-	42
^*^UV-A Activated ^*^ZnO/H_2_O_2_	8.17	9.77/f4.06	-	25
Proposed method	4	1.25/0.5	k=(2.6 ± 0.1)×l0^−2^ min^−1^	

Abbreviations: k, rate constant; UV-A, ultraviolet; ZnO, zinc oxide

**Table 4:** Comparison of the Studied Dyes with Other Dyes Using the Soybean Peroxidase Decoloration Method

**Dye**	**SBP concentration**	**pH**	**[H_2_O_2_] mM/[substrate] mM**	**Removal %**	**Reference**
Acid Blue 113	1.5 U/mL	4.0	2.5/1.0	95	32
Direct Black 38	0.75 U/mL	3.6	2.5/0.5	97	32
Remazol Turquoise Blue G 13	2.06×10^−7^ M	3.3	0.0998/0.18	96	43
Methyl Orange	0.373 U/mL	5.0	2/0.09	81	44
Crystal Ponceau 6R	0.27 μM	5	0.175/0.059	100	31
Trypan Blue	40 U/mL	4	0.064/0.045	90	30
**Proposed method**					

Acid Black 2	1.2 U/mL	4	1.25/0.5	95	
Acid Orange 7	1.2 U/mL	6.9	1.5/0.5	95	

The optimum pH depends on the proper ionization of the catalytic residues of SBP during the enzymatic reaction and may also depend on the type of dyes and their ionization states as a function of pH. Soybean peroxidase is active over a wide range of pH values (5.7–8.0).^35^ The pK_a_s of the functional groups in the dyes (i.e. phenolic OH 9.9, anilinium NH 4.6, naphtholic OH 9.3) could play a role in the optimum pH, especially with Acid Black 2.^45^,^46^ Similar pH optima were reported in previous studies using SBP in the removal of Trypan Blue dye (pH 4.0), a bisazo dye, Acid Blue 113 (pH 4.0), a bisazo dye, and Direct Black 38 (pH 3.6), a trisazo dye.^30^,^32^ Soybean peroxidase enzyme with a pH range of 3–5 demonstrated decoloration of Crystal Ponceau 6R, a monoazo dye (but only in the presence of a mediator), methyl orange, a monoazo dye, and Remazol Turquoise Blue G 133, a phthalocyanine dye.^31^,^43^,^44^ In one study, a pH range of 4–6 was reported optimal using horseradish peroxidase in the decolorization of Naphthol Blue Black, a bisazo dye, and in another study, pH 2 was optimum in the decoloration of Direct Yellow 106, a complex monoazo dye by Cucurbita pepper peroxidase.^47^,^48^ The optima recommended pH for further work are 4.4 and 6.9 for Acid Black 2 and Acid Orange 7, respectively. A sharp reduction in the percent conversion of Acid Orange 7 was observed in the slightly acidic region, possibly due to a change in the ionization of the catalytic residue of the enzyme in such conditions.^49^ The active site of soybean peroxidase contains an imidazole group of the distal histidine residue, and the pK_a_ of conjugate acid (protonated form) is 4.5.^50^

Soybean peroxidase requirements for degradation of some aromatic compounds were variable, as seen from previous studies: 0.6 U/mL for 1.0 mM aniline, 0.80 U/mL for 1.0 mM phenol, and 0.43 mU/mL for 0.10 mM benzidine.^27^,^35^,^51^ The same crude SBP as the present study required 0.75 and 1.5 U/mL for Acid Blue 113 and Direct Black 38, respectively, when prorated for the same dye concentration.^32^

The increased hydrogen peroxide concentration requirement over that predicted theoretically for dimer formation (0.5 H_2_O_2_/dye) is consistent with further polymerization. Further oligomerization can occur, as long as the oligomers remain soluble and contain the requisite phenolic and/or anilino functional groups, until the solubility limit is reached, thus its precipitation.^51^ The additional cycles of oligomerization require additional peroxide. Thus, the extra H_2_O_2_ consumed over the theoretical stoichiometric requirement is attributed to its consumption by the soluble dimeric and oligomeric compounds produced in the reaction. These results are consistent with earlier studies on the use of purified SBP in dye removal of Crystal Ponceau 6R.^31^ The same preparation of crude SBP required peroxide ratios of 2.5 and 5.0 for Acid Blue 113 and Direct Black 38, respectively.^32^

The rate of reaction should be directly proportional to the enzyme concentration; thus, the reaction would be expected to progressively slow down with the loss of enzyme activity over the reaction period, with loss of activity caused by reactive radicals and/or end-product oligomers.^37^ This is evident in Figure 5 where the actual progress curves lag behind the fitted curves beyond 60 min.^52^

## Conclusions

The present study found that crude SBP is a vital and effective catalyst for the decolorization and oxidative polymerization of hazardous aromatic azo dyes. The pH *(Figure 2),* hydrogen peroxide concentration *(Figure 4),* enzyme activity *(Figure 3)* and reaction time were key factors in the enzymatic treatment of the dyes. Limiting the amount of SBP resulted in lower substrate removal efficiencies, and excess peroxidase had no detrimental effect on the removal of the dyes *(Figure 3).* In addition, first-order fitting accounted reasonably well for the reaction progress curves *(Figure 5).*

## References

[1] Dellamatrice PM, Silva-Stenico ME, Moraes LA, Fiore MF, Monteiro RT (2017). Degradation of textile dyes by cyanobacteria. Braz J Microbiol.

[2] Sharma R, Singhal S (2015). Photodegradation of textile dye using magnetically recyclable heterogeneous spinel ferrites. J Chem Technol Biotechnol [Internet].

[3] Chan SH, Wu TY, Juan JC, Teh CY (2011). Recent developments of metal oxide semiconductors as photocatalysts in advanced oxidation processes (AOPs) for treatment of dye waste-water. J Chem Technol Biotechnol [Internet].

[4] Saratale RG, Saratale GD, Chang JS, Govindwar SP (2011). Bacterial decolorization and degradation of azo dyes: a review. J Taiwan Inst Chem Eng [Internet].

[5] Hassaan MA, El Nemr A, Madkour FF (2017). Testing the advanced oxidation processes on the degradation of Direct Blue 86 dye in wastewater. Egypt J Aquat Res [Internet].

[6] Gao S, Su J, Wang M, Wei X, Zheng X, Jiang T (2016). Electrochemical oxidation degradation of azobenzene dye self-powered by multilayer-linkage triboelectric nanogenerator. Nano Energy [Internet].

[7] Ercan O, Deniz S, Yetimoglu EK, Aydin A (2015). Degradation of reactive dyes using advanced oxidation method. Clean Soil Air Water [Internet].

[8] Das M, Bhattacharyya KG (2013). Oxidative degradation of Orange II dye in water with raw and acid-treated ZnO, and MnO2. Clean Soil Air Water [Internet].

[9] Presser C (2012). Absorption coefficient measurements of particle-laden filters using laser heating: validation with nigrosin. J Quant Spectrosc Radiat Transf [Internet].

[10] Eren Z (2012). Degradation of an azo dye with homogeneous and heterogeneous catalysts by sonophotolysis. Clean Soil Air Water [Internet].

[11] Adhikari S, Sarkar D (2015). Metal oxide semiconductors for dye degradation. Mater Res Bull [Internet].

[12] Borthakur P, Boruah PK, Darabdhara G, Sengupta P, Das MR, Boronin AI, Kibis LS, Kozlova MN, Fedorov VE (2016). Microwave assisted synthesis of CuS-reduced graphene oxide nanocomposite with efficient photocatalytic activity towards azo dye degradation. J Environ Chem Eng [Internet].

[13] Jo JO, Moon SH, Mok YS (2015). Non-thermal plasma degradation of dye using an underwater dielectric barrier discharge created inside a porous hydrophobic ceramic tube. Coloration Technol [Internet].

[14] Li D, Li J, Tang J (2016). Mercury oxide as an efficient photocatalyst for degradation of rhodamine B dye under visible-light irradiation. Solid State Sci [Internet].

[15] Zhang H, Wu J, Wang Z, Zhang D (2010). Electrochemical oxidation of Crystal Violet in the presence of hydrogen peroxide. J Chem Technol Biotechnol [Internet].

[16] Singh S, Lo SL, Srivastava VC, Hiwarkar AD (2016). Comparative study of electrochemical oxidation for dye degradation: parametric optimization and mechanism identification. J Environ Chem Eng [Internet].

[17] del Rio AI, Garcia C, Molina J, Fernandez J, Bonastre J, Cases F (2017). On the behavior of reduced graphene oxide based electrodes coated with dispersed platinum by alternate current methods in the electrochemical degradation of reactive dyes. Chemosphere [Internet].

[18] Dang TD, Banerjee AN, Tran QT, Roy S (2016). Fast degradation of dyes in water using manganese-oxide-coated diatomite for environmental remediation. J Phys Chem Solids [Internet].

[19] Lozovskyi O, Gunduz G, Dukkanci M, Prihod'ko R (2015). Preparation and characterisation of silver- or copper-doped TiO2 catalysts and their catalytic activity in dye degradation. Coloration Technol [Internet].

[20] Xu H, Yu T, Wang J, Li M, Liu Y (2015). Online monitoring of Fenton-mediated reactive red 6B oxidation kinetics. Environ Prog Sustain Energy [Internet].

[21] Castro E, Avellaneda A, Marco P (2014). Combination of advanced oxidation processes and biological treatment for the removal of benzidine-derived dyes. Environ Prog Sustain Energy [Internet].

[22] Shen Y, Xu Q, Wei R, Ma J, Wang Y (2017). Mechanism and dynamic study of reactive red X-3B dye degradation by ultrasonic-assisted ozone oxidation process. Ultrason Sonochem [Internet].

[23] Sekar S, Mahadevan S, Shanmugam BK, Mandal AB (2012). Bioenergetics and pathway of acid blue 113 degradation by Staphylococcus lentus. Biotechnol Prog [Internet].

[24] Sen SK, Raut S, Bandyopadhyay P, Raut S (2016). Fungal decolouration and degradation of azo dyes: a review. Fungal Biol Rev [Internet].

[25] Jaafar MT (2017). UV-A activated ZnO mediated photocatalytic decolorization of nigrosine (Acid Black 2) dye in aqueous solution. J Geosci Environ Prot [Internet].

[26] Zhang H, Wang W (2011). Oxidation of C.I. Acid Orange 7 with ozone and hydrogen peroxide in a hollow fiber membrane reactor. Chem Eng Commun [Internet].

[27] Mazloum S, Al-Ansari MM, Taylor K, Bewtra JK, Biswas N (2016). Additive effect on soybean peroxidase-catalyzed removal of anilines from water. Environ Eng Sci.

[28] Zaks A, Russell AJ (1988). Enzymes in organic solvents: properties and applications. Journal of Biotechnology.

[29] Feng W, Taylor KE, Biswas N, Bewtra JK (2013). Soybean peroxidase trapped in product precipitate during phenol polymerization retains activity and may be recycled. J Chem Technol Biotechnol [Internet].

[30] Kalsoom U, Ashraf SS, Meetani MA, Rauf MA, Bhatti HN (2013). Mechanistic study of a diazo dye degradation by soybean peroxidase. Chem Cent J [Internet].

[31] Ali L, Algaithi R, Habib HM, Souka U, Rauf MA, Ashraf SS (2013). Soybean peroxidase-mediated degradation of an azo dye–a detailed mechanistic study. BMC Biochem [Internet].

[32] Cordova Villegas LG, Mazloum S, Taylor KE, Biswas N (2018). Soybean peroxidase-catalyzed treatment of azo dyes with or without Feo pretreatment. Water Environ Res [Internet].

[33] Steevensz A, Cordova Villegas LG, Feng W, Taylor KE, Bewtra JK, Biswas N (2014). Soybean peroxidase for industrial wastewater treatment: a mini review. J Environ Eng Sci [Internet].

[34] Steevensz A, Madur S, Al-Ansari MM, Taylor KE, Bewtra JK, Biswas N (2013). A simple lab-scale extraction of soybean hull peroxidase shows wide variation among cultivars. Ind Crops Prod [Internet].

[35] Altahir BM, Feng W, Jasim HH, Taylor KE, Biswas N, Bewtra JK, Jassim SA (2015). Soybean peroxidase-catalysed removal of benzidines from water. J Environ Eng Sci [Internet].

[36] Stoll VS, Blanchard JS, Burgess RR, Deutscher MP (2009). Buffers: principles and practice. Methods in enzymology [Internet].

[37] Al-Ansari MM, Steevensz A, Taylor KE, Bewtra JK, Biswas N (2010). Soybean peroxidase-catalyzed removal of an aromatic thiol, 2-mercaptobenzothiazole, from water. Water Environ Res [Internet].

[38] Bolova E, Gunduz G, Dukkanci M (2012). Heterogeneous Fenton-like degradation of Orange II in water using FeZSM-5 zeolite catalyst. Int J Chem React Eng [Internet].

[39] Tabai A, Bechiri O, Abbessi M (2017). Degradation of organic dye using a new homogeneous Fenton-like system based on hydrogen peroxide and a recyclable Dawson-type heteropolyanion. Int J Ind Chem [Internet].

[40] Zhang H, Fu H, Zhang D (2009). Degradation of C.I. Acid Orange 7 by ultrasound enhanced heterogeneous Fenton-like process. J Hazard Mater [Internet].

[41] Zhong X, Xiang L, Royer S, Valange S, Barrault J, Zhang H (2011). Degradation of C.I. Acid Orange 7 by heterogeneous Fenton oxidation in combination with ultrasonic irradiation. J Chem Technol Biotechnol [Internet].

[42] Akash T, Gidde MR (2014). Decolourization of Nigrosine WS (AB2) Dye by Solar Photo-Fenton Process. Int J Sci Technol [Internet].

[43] Marchis T, Avetta P, Bianco-Prevot A, Fabbri D, Viscardi G, Laurenti E (2011). Oxidative degradation of Remazol Turquoise Blue G 133 by soybean peroxidase. J Inorg Biochem [Internet].

[44] Chiong T, Lau SY, Lek ZH, Koh BY, Danquah MK (2016). Enzymatic treatment of methyl orange dye in synthetic wastewater by plant-based peroxidase enzymes. J Environ Chem Eng [Internet].

[45] Silverstein TP, Heller ST (2017). pKa values in the undergraduate curriculum: what is the real pKa of water?. J Chem Educ [Internet].

[46] de Lange AM, Potgieter JH (1991). Acid and base dissociation constants of water and its associated ions. J Chem Educ [Internet].

[47] Onder S, Celebi M, Altikatoglu M, Hatipoglu A, Kuzu H (2011). Decolorization of naphthol blue black using the horseradish peroxidase. Appl Biochem Biotechnol [Internet].

[48] Boucherit N, Abouseoud M, Adour L (2013). Degradation of direct azo dye by Cucurbita pepo free and immobilized peroxidase. J Environ Sci (China) [Internet].

[49] Al-Ansari MM, Steevensz A, Al-Aasm N, Taylor KE, Bewtra JK, Biswas N (2009). Soybean peroxidase-catalyzed removal of phenylenediamines and benzenediols from water. Enzym Microb Technol [Internet].

[50] Kamal JK, Behere DV (2002). Thermal and conformational stability of seed coat soybean peroxidase. Biochem [Internet].

[51] Caza N, Bewtra JK, Biswas N, Taylor KE (1999). Removal of phenolic compounds from synthetic wastewater using soybean peroxidase. Water Res [Internet].

[52] Rahman K (2007). Studies on free radicals, antioxidants, and co-factors. Clin Interv Aging [Internet].

